# Evaluation of anti-inflammatory and ulcerogenic potential of zinc–ibuprofen and zinc–naproxen complexes in rats

**DOI:** 10.1007/s10787-017-0361-0

**Published:** 2017-05-23

**Authors:** Magdalena Jarosz, Natalia Szkaradek, Henryk Marona, Gabriel Nowak, Katarzyna Młyniec, Tadeusz Librowski

**Affiliations:** 1 Department of Radioligands, Jagiellonian University Medical College, Medyczna 9, 30-688 Krakow, Poland; 2 Department of Bioorganic Chemistry, Chair of Organic Chemistry, Jagiellonian University Medical College, Medyczna 9, 30-688 Krakow, Poland; 3 Department of Pharmacobiology, Jagiellonian University Medical College, Medyczna 9, 30-688 Krakow, Poland; 4 Institute of Pharmacology, Polish Academy of Sciences, Smetna 12, 31-343 Krakow, Poland

**Keywords:** Zinc, NSAIDs, Zinc complexes, Anti-inflammatory activity, Gastric ulcers

## Abstract

Because of numerous indications and high availability, non-steroidal anti-inflammatory drugs (NSAIDs) are among the most commonly prescribed and used medicines in the world. However, long-term therapy with and improper use of NSAIDs may lead to gastrointestinal damage. Therefore, improving the therapeutic index of the existing drugs has become a priority over the past decades. Considerable attention in the field has been concentrated on metal complexes of non-steroidal anti-inflammatory drugs. The aim of this study is to evaluate the effect of complexation with zinc on the anti-inflammatory and ulcerogenic effects of ibuprofen and naproxen after single and triple intragastric administration to rats. The anti-inflammatory effect was assessed in carrageenan-induced inflammatory edema in the hind paw of male albino Wistar rats. The mucosal lesions were inspected and evaluated for gross pathology. Single administration of both the investigated complexes, namely zinc–ibuprofen and zinc–naproxen (20 mg/kg equivalent to ibuprofen and naproxen, respectively) and their parent drugs and physical mixtures with zinc hydroaspartate (ZHA doses: 16.05 and 14.37 mg/kg), caused a significant reduction of the edema after the same time from the carrageenan injection in comparison to the control groups. However, no statistically significant differences between the investigated drugs were observed after their single administration. The mean ulceration score for the mixture of ibuprofen and ZHA was statistically lower than the mean score achieved in rats after treatment with ibuprofen alone. On the other hand, triple intragastric administration of the ZHA–ibuprofen and ZHA–naproxen combination showed substantial enhancement of the anti-inflammatory activity against control groups, as well as against the parent NSAIDs. The most potent anti-inflammatory activity was demonstrated after 2 h from the carrageenan injection in animals receiving ZHA together with naproxen. The edema growth was reduced in these animals by 80.9% as compared to the control group. This result was significantly higher than the results achieved in animals receiving zinc–naproxen (50.2%) or naproxen alone (47.9%). Both NSAID complexes with zinc and mixtures with ZHA alleviated ulcerations caused by parent NSAIDs; however, the mixtures of both ibuprofen and naproxen with ZHA after triple administration were the least damaging. In view of the above results, zinc supplementation during NSAID therapy may have a beneficial effect on ulcer prevention and healing by reducing the effective dose of the parent drug and increasing its potency.

## Introduction

**Fig. 1:**
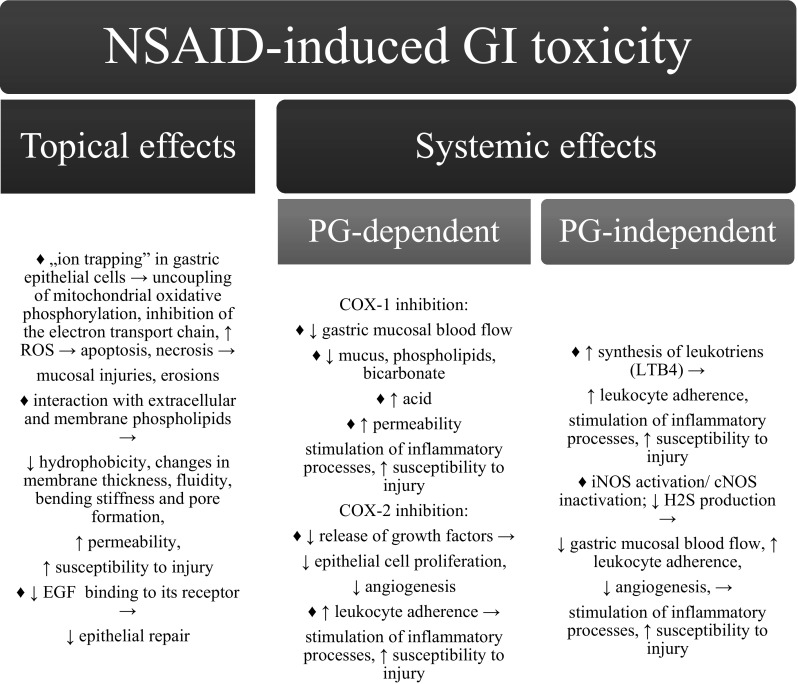
Mechanisms of NSAID-induced GI toxicity can be divided into topical and systemic effects, and the latter may be prostaglandin-dependent through COX inhibition or prostaglandin-independent (Wallace 2008; Lim et al. 2009; Musumba et al. 2009)

Since the ancient times, medicines derived from willow trees and other salicylate-rich plants have been used for relieving pain and reducing fever. However, it was not until the isolation of salicin in 1828 and the subsequent development of the drug aspirin in 1899 that pharmacologists’ interest in non-steroidal anti-inflammatory drugs (NSAIDs) was awakened. Currently, because of numerous indications and high availability, NSAIDs are among the most commonly prescribed and used medicines in the world (Patrono and Rocca 2009; Markiewicz and Pasenkiewicz-Gierula 2011). The primary mechanism of action of NSAIDs is the inhibition of cyclooxygenase (COX) enzymes, leading to reduced prostaglandin biosynthesis, which determines their versatile effectiveness as analgesics, antipyretics, and anti-inflammatory agents (Capone et al. 2007). Unfortunately, this mechanism also largely contributes to the gastrointestinal (GI) toxicity of NSAIDs (Watanabe et al. 2002; Wallace 2008). The most common side effects during NSAID therapy include gastric and duodenal ulcerations and bleedings. Further, the small intestine and the colon are exposed to numerous complications including bleeding, perforation, stricture, and chronic problems, such as iron deficiency anemia and protein loss (Sostres et al. 2010; Park et al. 2011). The inhibited synthesis of cytoprotective prostaglandins (PGE_2_ and PGI_2_) increases gastric acid secretion, reduces mucus synthesis and bicarbonate secretion, and impairs gastric mucosal blood flow. Consequently, gastric mucosal defense and healing are severely impaired (Wallace 2008). Irrespective of how remarkable the correlation between the suppressed PG synthesis and the occurrence of ulcers is, the pathogenesis of NSAID-induced ulcerations is complex (Lim et al. 2009; Musumba et al. 2009). Recent findings have shown that factors such as nitric oxide and hydrogen sulfide, growth factors, neuropeptides, hormones, and stress proteins act in concert with PGs in maintaining the gastric mucosal defense. Moreover, the direct local effect of acidic NSAIDs plays a significant role in ulcer formation. Factors contributing to NSAID-induced GI toxicity are summarized in Fig. 1.

Recent approaches to reduce GI side effects during NSAID therapy are based on the introduction of potential novel therapeutics, such as nitric oxide (NO) or hydrogen sulfide (H_2_S)-complexed NSAIDs, lipid-modified NSAIDs, and metal-complexed NSAIDs (Lim et al. 2009; Fiorucci and Distrutti 2011). The transport of organic ligands into the cells can be facilitated by the formation of metal complexes (Sharma et al. 2003; Abu Ali et al. 2015). Therefore, NSAIDs complexation with metals, particularly copper and zinc, may yield measurable benefits, i.e., reduce the effective dose of the parent drug and increase potency, while limiting adverse reactions, expanding indications for use, or increasing the selectivity toward certain diseases (Dillon et al. 2003).

Zinc has been shown to modulate immune response and may be considered an important agent with anti-inflammatory and antioxidant activity (Jarosz et al. 2017). The role of zinc in accelerating the healing of wounds, including ulcers, is also recognized worldwide (Lansdown et al. 2007). In 1994, the first gastroprotective chelate of zinc and l-carnosine (polaprezinc, PZ) was introduced into the Japanese market. Although this drug has already been in use for more than 20 years, its mechanism of action remains not fully understood. The gastroprotection exhibited by PZ has been attributed to its antioxidant activity, the stabilizing effect on membranes, the inducing effect on mucus and endogenous PG synthesis, and the stimulating effect on heat shock proteins HSP 70 and HSP 32 [heme oxygenase (HO)-1], recognized as potential gastroprotective agents (Shimada et al. 1999; Naito et al. 2001; Ueda et al. 2009; Opoka et al. 2010). Some of the studies have shown the key role played by zinc in the inhibition of indomethacin-induced apoptosis of gastric mucosa by inhibiting the activity of caspase-3 (the same study did not confirm the anti-apoptotic properties of carnosine) (Fuji et al. 2000). It was also demonstrated that PZ inhibits the expression of mRNA for TNF-α, reduces its levels in the gastric mucosa, and inhibits the activation of NF-kB (Shimada et al. 1999; Naito et al. 2001). Moreover, zinc sulfate has been found to prevent indomethacin-induced changes in the content of mucosal lipids and sugars followed by the translocation of luminal bacteria (Sivalingam et al. 2011). In accordance to recently conducted research, treatment with zinc hydroaspartate (ZHA) stimulates gastric microcirculation, significantly reduces gastric secretion, and increases the plasma gastrin level. The administration of ZHA after the induction of ulcers in animals (ZHA dose: 65 mg/kg daily) resulted in an increase in the zinc ion levels in the gastric juice and the ulcerated area, which significantly accelerated the healing process at day 7 upon ulcer induction (Opoka et al. 2010).

The anti-inflammatory, antioxidant, and wound healing properties of zinc became the basis for the idea of its complexation with NSAIDs. Selected results of the thus far tested complexes are summarized in Table 1.

**Table 1:** Results of hind paw edema test and evaluation of ulcerogenic effect of zinc complexes with selected NSAIDs

NSAID complex (references)	Treatment	Animals	Scheme of hind paw edema test	Results of hind paw edema test	Ulcerogenic effect (evaluated separately from hind paw edema test)
Zn-Indomethacin (Singla and Wadhwa 1995)	Zn-Ind doses equivalent to 1.5–12 mg/kg of indomethacin in water p.o.	Wistar 100–150 g *n* = min 6	−1 h (NSAID administration prior to carrageenan administration) 0.5 h/1 h/1.5/2 h/3 h (measurements after carrageenan administration)	Zn-Ind was 3 × more potent than Ind	No significant reduction of gastric ulcers Zn-Ind vs. Ind
Zn-Diclofenac (Santos et al. 2004)	10 mg/kg in 0.5% solution of CMC p.o.	Wistar 180–300 g *n* = 6	−2 h 2 h/4 h/6 h	No significant differences Zn-Dic vs. Dic	Significant reduction of ulcers Zn-Dic vs. Dic
Zn-Naproxen (Sharma et al. 2003)	100 mg/kg in solution of CMC p.o.	Wistar 100–200 g *n* = min 6	−0.5 h 0.5 h/1 h/2 h/3 h/4 h	Significant differences Zn-Nap vs. Nap	Significant reduction of ulcers LI C/Nap/Zn-Nap 0.5/10/4
Zn-Naproxen (Jain et al. 1999)	4–16 mg/kg in 0.5% solution of CMC p.o.	Portan rats 200–250 g *n* = 6	−0.5 h 1 h/2 h/3 h/4 h	No significant differences Zn-Nap vs. Nap	Significant reduction of ulcers LI C/Nap/Zn-Nap 0/4.5–7.0/1.5–2.7
Zn-Piroxicam (Tagliati et al. 1999)	10 mg/kg in 0.5% solution of CMC p.o.	Wistar 170–190 g *n* = 8	−0.5 h 1 h/2 h/3 h/4 h/5 h/24 h	No significant differences Zn-Pir vs. Pir	Significant reduction of ulcers LI C/Pir/Zn-Pir 31.9/59.3/45.6

*CMC* carboxymethylcellulose, *LI* lesion index, *C* control

The study’s aim was to evaluate the effect of complexation with zinc on the anti-inflammatory and ulcerogenic effects of ibuprofen and naproxen after single and triple intragastric administration to rats.

## Materials and methods

### Materials

#### Animals

Male albino Wistar rats, weighing between 155 and 205 g, were used for the anti-inflammatory tests. The animals were housed and fed in a laboratory and kept at a constant temperature of 22 °C under standard conditions (a 12:12-h L:D cycle, standard pellet diet, tap water). Treatment of laboratory animals in the present study was in full accordance with the respective Polish and European regulations date and file or reference number of Ethic's Committee approval of the animal study.

#### Chemicals

Zinc–ibuprofen and zinc–naproxen complexes were synthesized in the Department of Bioorganic Chemistry, Chair of Organic Chemistry, Faculty of Pharmacy, Jagiellonian University Medical College. Both the synthesized complexes, as well as ibuprofen (Sigma-Aldrich, Germany), naproxen (Sigma-Aldrich, Germany), and ZHA (Farmapol, Poznań) for intragastric administration were finely powdered and suspended/dissolved ex tempore in distilled water.

## Methods

### Preparation of zinc complexes of ibuprofen and naproxen

Sodium hydroxide (0.1 mol NaOH) was dissolved in the smallest amount of H_2_O. To this solution, 0.1 mol of naproxen was added with constant stirring. Complete dissolution meant sodium naproxen formation. Then, 0.07 mol of ZnSO_4_·7H_2_O dissolved in the smallest amount of H_2_O was added with constant stirring. The precipitated zinc–naproxen complex was filtered and washed with NaHCO_3_ and then with H_2_O to remove the unreacted naproxen. The obtained filtrate was acidified to recover the unreacted naproxen. The same procedure was used for obtaining the zinc–ibuprofen complex.

Determination of anti-inflammatory activity of the investigated compounds using the carrageenan-induced hind paw edema test.

Animals that fasted for 24 h before the experiment were used in the hind paw oedema test. Rats were randomly divided into five groups, each with seven individuals. The experiments were conducted according to the approved scheme (Table 2). The tested compounds were administered intragastrically to the fasted rats having free access to drinking water. After 1 h, to produce inflammation, 0.1 mL of 1% carrageenan solution in water was injected into the hind paw subplantar tissue of rats, according to the modified method of Winter et al. (1962). The development of paw edema was measured plethysmographically (Ugo Basile, Italy). Paw diameters were measured and recorded prior to the carrageenan injection and after 1, 2, and 3 h, while the percentage of the edema inhibition was calculated according to the following formula:

$${\text{Oedema}}\;{\text{inhibition}}\;\% = \frac{{(N - N^{{\prime }} \times 100) }}{N},$$

where *N* denotes the paw diameters measured 1, 2, and 3 h after the carrageenan injection to the control group—paw diameters at the beginning. *N*′ represents the paw diameters measured 1, 2, and 3 h after the carrageenan injection to the test groups—paw diameters at the beginning.

**Table 2:** Scheme of the experiments

	Single intragastric administration 1 × p.o.	Triple intragastric administration 3 × p.o.
Zn-ibuprofen *n* = 7	1. Control (water p.o.) 2. ZHA 16.05 mg/kg in water (zinc 3.17 mg/kg) 3. Ibuprofen 20 mg/kg in water 4. Zn-ibuprofen 24.93 mg/kg in water (ibuprofen 20 mg/kg and zinc 3.17 mg/kg) 5. ZHA 16.05 mg/kg + Ibuprofen 20 mg/kg in water	Rats divided into five groups (according to the scheme of single administration) received investigated compounds for three consecutive days. Prior to third administration rats were fasted for 24 h. The hind paw edema test was performed on fasted rats on day 3. Rats were killed on day 4
Zn-naproxen *n* = 7	1. Control (water p.o.) 2. ZHA 14.37 mg/kg in water (zinc 2.84 mg/kg) 3. Naproxen 20 mg/kg in water 4. Zn-naproxen 24.41 mg/kg in water (ibuprofen 20 mg/kg and zinc 2.84 mg/kg) 5. ZHA 14.37 mg/kg + Naproxen 20 mg/kg in water	Rats divided into five groups (according to the scheme of single administration) received investigated compounds for three consecutive days. Prior to third administration rats were fasted for 24 h. The hind paw edema test was performed on fasted rats on day 3. Rats were killed on day 4

### Irritant action on the gastric mucosa according to Komatsu

The ulcerogenic effect was determined by the method of Komatsu et al. (1973). The tested compounds were administered to the fasted rats having free access to drinking water. Twenty-four hours after the administration of the compounds, the rats were killed, and their stomachs were removed and after incision along the lesser curvature, rinsed with a tap soaked in warm (37 °C) saline, spread on a cork board, and pinned down. The mucosa of the glandular part of the stomach was inspected using a binocular microscope (tenfold magnification). The stomachs were photographed. The mucosal lesions were evaluated using a 0–5 point scale (0: no lesions, 1: erythema, 2: hemorrhagic streaks, 3: small ulcers, 4: large ulcers, 5: perforation).

### Statistical analysis

In the carrageenan-induced hind paw edema test, the obtained data were evaluated by a two-way analysis of variance (two-way ANOVA), followed by Bonferroni’s multiple comparison test; *p* < 0.05 was considered significant. In ulcerogenic activity the test data were evaluated by a two-way analysis of variance (two-way ANOVA), followed by Tukey’s test; *p* < 0.05 was considered significant.

## Results and discussion

**Fig. 2:**
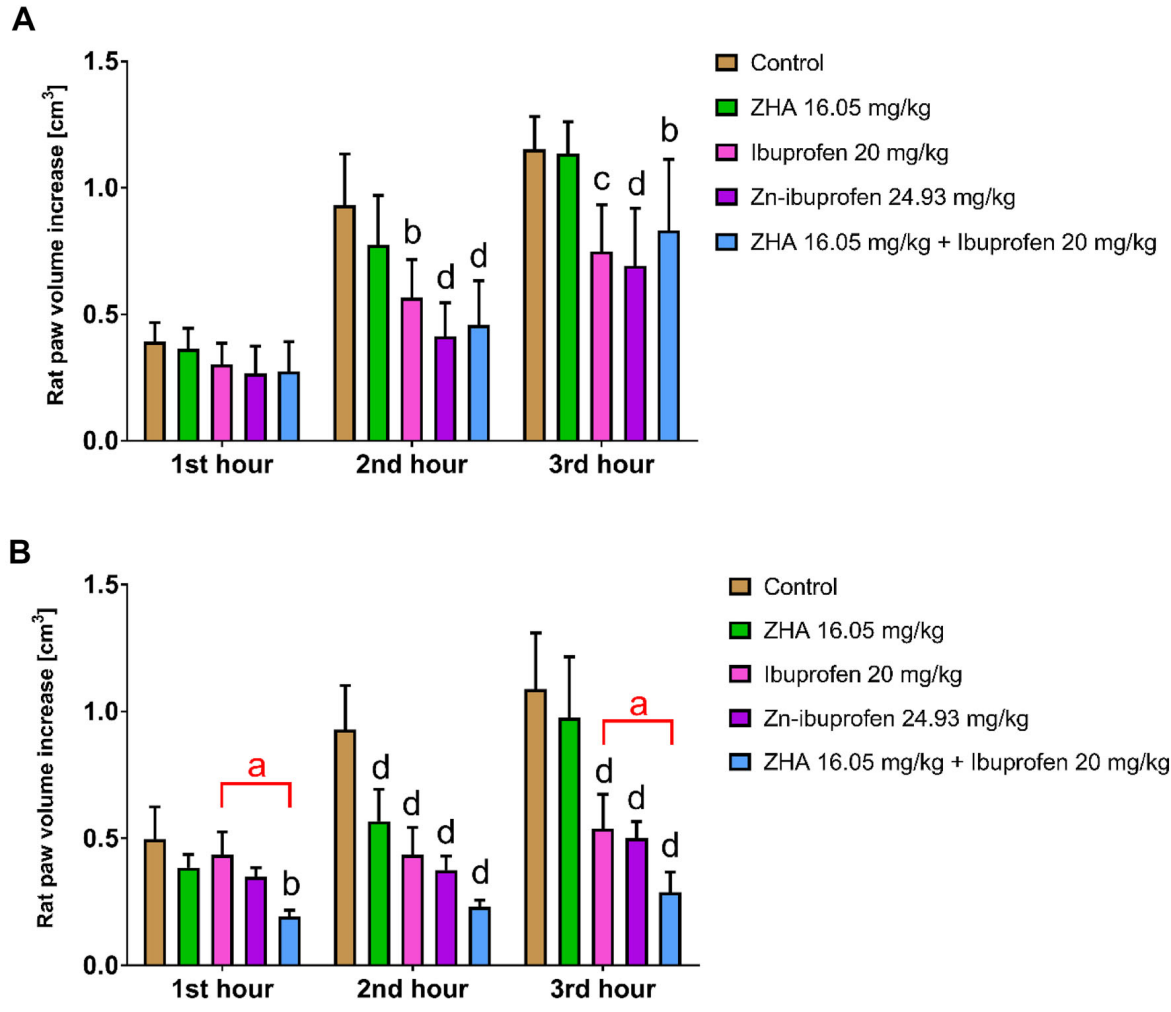
Results of carrageenan-induced hind paw edema test for zinc hydroaspartate (ZHA), ibuprofen, zinc–ibuprofen, and physical mixture of ZHA and ibuprofen after their single (**a**) and triple (**b**) administration. Data expressed as the mean ± SD; evaluated by a two-way analysis of variance (two-way ANOVA), followed by Bonferroni’s multiple comparison test; ^a^ *p* < 0.05, ^b^ *p* < 0.01, ^c^ *p* < 0.001, and ^d^ *p* < 0.0001; *n* **=** 6–7

**Fig. 3:**
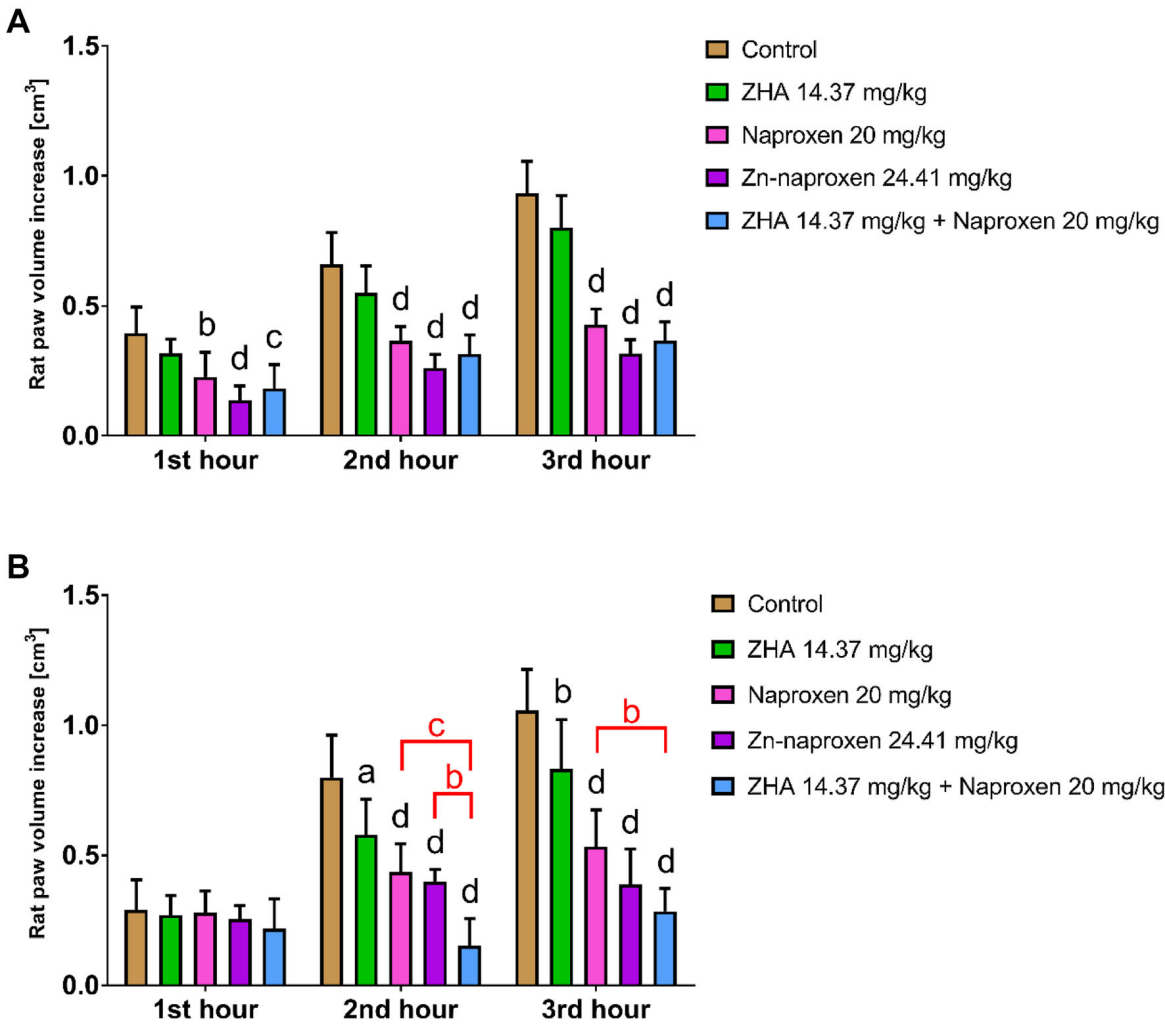
Results of carrageenan-induced hind paw edema test for zinc hydroaspartate (ZHA), naproxen, zinc–naproxen, and physical mixture of ZHA and naproxen after their single (**a**) and triple (**b**) administration. Data expressed as the mean ± SD; evaluated by a two-way analysis of variance (two-way ANOVA), followed by Bonferroni’s multiple comparison test; ^a^ *p* < 0.05, ^b^ *p* < 0.01, ^c^ *p* < 0.001, and ^d^ *p* < 0.0001; *n* **=** 6–7

The results of the carrageenan-induced hind paw edema test of Zn–ibuprofen and Zn–naproxen are shown in Figs. 2 and 3 and the percentage of edema inhibition are presented in Tables 3 and 4, respectively.

**Table 3:** Results of hind paw oedema test expressed as a percentage

	Percent of oedema inhibition		
	1st hour	2nd hour	3rd hour
1 × p.o.			
ZHA	7.0	16.7	1.5
Ibuprofen	22.7	**39.2** ^b^	**35.1** ^c^
Zn-ibuprofen	31.8	**55.5** ^d^	**39.9** ^d^
ZHA + Ibuprofen	29.6	**50.7** ^d^	**27.9** ^b^
3 × p.o.			
ZHA	22.5	**36.6** ^d^	10.4
Ibuprofen	12.4	**51.5** ^d^	**50.5** ^d^
Zn-ibuprofen	29.9	**58.2** ^d^	**54.1** ^d^
ZHA + Ibuprofen	***61.3*** ^b^	**74.4** ^d^	***73.5*** ^d^

Bold font indicates results that were statistically significant vs. the control: ^a^ *p* < 0.05, ^b^ *p* < 0.01, ^c^ *p* < 0.001, and ^d^ *p* < 0.0001. Bold italic font indicates results that were statistically significant vs. the parent NSAID

**Table 4:** Results expressed as a percentage

	Percent of oedema inhibition		
	1st hour	2nd hour	3rd hour
1 × p.o.			
ZHA	19.4	16.7	14.1
Naproxen	**42.5** ^b^	**44.6** ^d^	**54.1** ^d^
Zn-naproxen	**65.6** ^d^	**60.4** ^d^	**66.0** ^d^
ZHA + Naproxen	**53.7** ^c^	**52.2** ^d^	**60.7** ^d^
3 × p.o.			
ZHA	6.9	**27.5** ^a^	**21.2** ^b^
Naproxen	13.3	**47.9** ^d^	**49.2** ^d^
Zn-naproxen	11.8	**50.2** ^d^	**63.4** ^d^
ZHA + Naproxen	25.1	***80.9*** ^d^	***73.1*** ^d^

Bold font indicates results that were statistically significant vs. the control: ^a^ *p* < 0.05, ^b^ *p* < 0.01, ^c^ *p* < 0.001, and ^d^ *p* < 0.0001. Bold italic font indicates results that were statistically significant vs. the parent NSAID

Single intragastric administration of ZHA alone in doses of 16.05 and 14.37 mg/kg did not significantly influence the rat hind paw edema. This result may be attributed to the low doses of zinc used (zinc ions in the amount of 3.17 and 2.84 mg/kg). In contrast, triple intragastric administration of the same low doses of zinc significantly reduced the edema 2 h after the carrageenan injection by 36.6% (ZHA dose: 16.05 mg/kg) and after 2 and 3 h, by 27.5 and 21.2%, respectively (ZHA dose: 14.37 mg/kg). Zinc exerts its anti-inflammatory and antioxidant activity through several acute and chronic mechanisms (Powell 2000). It acts by antagonizing transition metal-catalyzed reactions, stabilizing the protein sulfhydryls, activating antioxidant proteins, enzymes, and metallothioneins. Moreover, zinc decreases NF-κB activation and its target genes, such as TNF-α and IL-1β, and increases the gene expression of A20 and PPAR-α, the two zinc finger proteins with anti-inflammatory properties (Jarosz et al. 2017). Repeated administration of low doses of ZHA in both experiments did confirm the anti-inflammatory activity exerted by zinc ions.

Anti-inflammatory studies using carrageenan-induced hind paw edema showed differences in the anti-inflammatory activity of the parent NSAIDs, their zinc complexes, and the physical mixtures of NSAIDs and ZHA. Single administration of both investigated complexes, zinc–ibuprofen and zinc–naproxen, as well as their parent drugs and physical mixtures with ZHA, caused statistically significant reduction of the edema after the same time from the carrageenan injection in comparison to the control groups. The highest percentage of edema inhibition was observed for Zn–NSAID complexes; zinc–ibuprofen reduced the edema after 2 and 3 h by 55.5 and 39.9%, whereas zinc–naproxen reduced the edema after 2 and 3 h by 60.4 and 66.0%, respectively. No statistically significant differences between the investigated drugs were observed after their single intragastric administration.

Only 3-day experiments revealed significant differences in the anti-inflammatory activity between the investigated compounds. The mixture of ibuprofen and ZHA significantly reduced the edema after 1 and 3 h from the carrageenan injection in comparison to ibuprofen alone (61.3 and 73.5 vs. 12.4 and 50.5%, respectively). The differences between zinc–ibuprofen and the mixture of ZHA and ibuprofen were insignificant; however, the achieved percentage of edema inhibition was higher for the mixture. A similar trend was observed for the triple intragastric administration of naproxen, its complex with zinc, and the physical mixture with ZHA. The most potent anti-inflammatory activity was demonstrated after 2 h from the carrageenan injection in animals receiving ZHA together with naproxen. The edema development was reduced in these animals by 80.9% as compared to the control group. This result was significantly higher than the results achieved in animals receiving zinc–naproxen (50.2%) or naproxen alone (47.9%). The mixture remarkably reduced the edema also after 3 h from the carrageenan injection (the result was significantly higher than the result obtained in the naproxen group). Repeated intragastric administration of the ZHA–ibuprofen and ZHA–naproxen combinations showed a substantial enhancement of the anti-inflammatory activity against ibuprofen and naproxen, respectively.

These results are in line with the ulcerogenic effects determined according to the method established by Komatsu et al. (1973). The results of irritant action on the gastric mucosa are listed in Tables 5 and 6. The ulceration scores for the control and the ZHA animals were equal to zero and are not included in the tables. Both NSAID complexes with zinc and the mixtures with ZHA alleviated ulcerations caused by the parent NSAIDs. The differences in the ulcerogenic potential between the complexes and the mixtures were negligible; however, the mixtures of both ibuprofen and naproxen with ZHA after the triple administration were the least damaging. Moreover, the mean ulceration score for the mixture of ibuprofen and ZHA after the single administration was significantly lower than the mean score achieved in rats after treatment with ibuprofen alone. To the best of our knowledge, this is the first report comparing the anti-inflammatory and ulcerogenic effects of the zinc–ibuprofen and zinc–naproxen complexes after their single and triple administration to rats. Furthermore, we collated these effects with the results obtained after the administration of not only the parent NSAIDs but also their mixtures with ZHA and ZHA alone.

**Table 5:** Irritant action on the gastric mucosa according to Komatsu

	Ulcerogenic effect	
	Single intragastric administration 1 × p.o.	Triple intragastric administration 3 × p.o.
Ibuprofen	1.67 ± 0.11	1.58 ± 0.08
Zn-ibuprofen	1.08 ± 0.30	1.33 ± 0.11
ZHA + Ibuprofen	**1.00** ^a^ ± 0.13	1.00 ± 0.22

The mucosal lesions were evaluated using a 0–5 point scale (0: no lesions, 1: erythema, 2: hemorrhagic streaks, 3: small ulcers, 4: large ulcers, 5: perforation). Data evaluated by a two-way analysis of variance (two-way ANOVA), followed by Tukey’s test. Each result is an average of six rates ± SEM. Bold font indicates results that were statistically significant vs. the parent NSAID: ^a^ *p* < 0.05

**Table 6:** Irritant action on the gastric mucosa according to Komatsu

	Ulcerogenic effect	
	Single intragastric administration 1 × p.o.	Triple intragastric administration 3 × p.o.
Naproxen	1.92 ± 0.15	1.67 ± 0.17
Zn-naproxen	1.75 ± 0.11	1.50 ± 0.18
ZHA + Naproxen	1.83 ± 0.11	1.42 ± 0.15

The mucosal lesions were evaluated using a five-point scale (0: no lesions, 1: erythema, 2: punctiform ulcers, 3: small ulcers, 4: large ulcers, 5: perforation). Data evaluated by a two-way analysis of variance (two-way ANOVA), followed by Tukey’s test. Each result is an average of six rates ± SEM

The search for more effective and less toxic anti-inflammatory drug treatments continues. Several earlier studies were designed to characterize transition metal complexes of NSAIDs and evaluate their anti-inflammatory, antioxidant, antibacterial, or ulcerogenic activity. The results of the anti-inflammatory and ulcerogenic potential of the thus far tested zinc complexes of NSAIDs are summarized in Table 1. In the study by Singla and Wadhwa (1995), the zinc–indomethacin complex was three times more potent as an anti-inflammatory agent than pure indomethacin. However, no statistically significant difference in the lesion index between the complex and an equivalent amount of indomethacin was observed. The authors seek the cause of such observation in the extensive enterohepatic circulation of indomethacin in the bile, as well as in the very small content of zinc (0.9 mg/kg) present in the zinc–indomethacin complex. Consequently, the contribution of zinc toward an increase in the anti-inflammatory potency seems to be minimal, and this increase is attributed by the authors mainly to a greater rate and extent of absorption of the indomethacin from the complexed form. On the other hand, studies by Santos et al. (2004), Jain et al. (1999), and Tagliati et al. (1999) demonstrated that the anti-inflammatory activity of diclofenac, naproxen, and piroxicam were not changed by complexing with zinc, whereas a reduction of the severity of lesions was observed in all the three experiments. In contrast to the results achieved by Jain et al. (1999), other group have shown a statistically significant difference in the anti-inflammatory activity of naproxen and its zinc complex (Sharma et al. 2003). Furthermore, a significant reduction in the lesion index of the zinc complex was observed as compared to naproxen. Nevertheless, the selected doses of naproxen and its zinc complex were considerably higher (100 mg/kg equivalent to naproxen) than doses tested by other researchers.

In contrast to earlier opinions that the metal ion complexation of organic ligands facilitates their transport into the cells, Tagliati et al. (1999) observed that the absorption of the zinc–piroxicam complex was slower than the absorption of the free drug. In the opinion of the authors, the slower, progressive absorption pattern may contribute to the reduction of adverse effects in a manner similar to slow-release pharmaceutical formulations. At the same time, no statistically significant differences in the anti-inflammatory activity between piroxicam and zinc–piroxicam were observed; paw increases were similar in value after 1, 2, and 5 h from the carrageenan injection. We suppose that zinc may have contributed to the anti-inflammatory effect exerted by zinc–piroxicam during first 2 h after the carrageenan injection, as anti-edematous effects of both the complex and piroxicam were similar and the plasma piroxicam levels were lower after the administration of the complex than after that of piroxicam.

In our experiments, the doses of the investigated NSAIDs and their zinc complexes were selected on the basis of the earlier dose-dependence studies (our unpublished data). The dosage and the salt form of zinc were chosen with respect to previous research. For example, in the study by Sharma et al. (2003), different doses of naproxen and its zinc complex were used for evaluating the anti-inflammatory and ulcerogenic potential of the investigated compounds; i.e., naproxen and zinc–naproxen in the hind paw edema test were used in the dose of or equivalent to 100 mg/kg, whereas the ulcerogenic effect was determined for a dose of 29 mg/kg administered to rats twice a day over a 2-day period. Further, the dose of zinc in the form of zinc sulfate was remarkably higher (fivefold higher) than doses applied in our experiments; the single dose of zinc ions used in the hind paw edema test was 15 mg/kg. The authors perceived the reasons for the damage to the gastric mucosa caused by the mixture of naproxen and zinc sulfate to be the ulcerogenic effect of naproxen along with the corrosive effects of an overdosage of zinc sulfate on the gastric mucosa, which, by its conversion to zinc chloride in the stomach, is highly astringent. Therefore, in our experiments, instead of inorganic zinc salts, we administered to the rats ZHA, which was proven by Opoka et al. (2010) to possess gastroprotective potential. Moreover, as the standard rat diet contains a sufficient amount of zinc to maintain the animals’ health and no pathological conditions were induced in rats before the drugs’ administration, we reduced the risk of overdosing zinc ions by selecting low doses of the zinc salt. To facilitate comparisons, we used two uneven doses of zinc in the form of ZHA (16.05 and 14.37 mg/kg), which were equivalent to the doses of zinc contained in the zinc complexes.

According to Mohod and Bodhankar (2013), naproxen administered at a dose of 30 mg/kg p.o. consecutively for 3 days clearly showed a gastric antral ulcer and dramatically decreased superoxide dismutase (SOD), glutathione (GSH), and nitric oxide (NO), as well as increased malondialdehyde (MDA), myeloperoxidase (MPO), and histamine in the rats’ stomachs. Researchers perceive the major underlying factor of naproxen-induced gastric antral ulceration in the generation of oxygen-free radicals and lipid peroxidation. Moreover, other studies indicate the significant role of enzymes such as SOD, catalase (CAT), and glutathione peroxidase (GPx) in the defense against the oxidative tissue damage of the gastric mucosa after the administration of naproxen (Kim et al. 2005, 2014). Moreover, in the rat model, the nuclear factor erythroid 2-related factor 2 (Nrf2) expression in the naproxen-induced gastric ulcer group was lower than in the untreated rats, suggesting that Nrf2 plays an important role in naproxen-induced gastric ulceration and its subsequent alleviation (Kim et al. 2014). Zinc is a co-factor of the cytosolic and extracellular Zn/Cu SOD enzyme, which acts as an ROS scavenger and contributes to the regulation of neutrophil apoptosis and neutrophil-mediated tissue injury (Yasui et al. 2005; Yasui and Baba 2006; Mariani et al. 2008). Moreover, zinc treatment to chlorpyriphos-intoxicated animals elevated the levels of GSH, CAT, and detoxifying glutathione-S-transferase (GST) (Goel et al. 2005). Nrf2, the critical transcription factor that regulates the expression of genes encoding the above-mentioned antioxidant and detoxifying molecules (GSH, SOD, and GST) has been proven to be up-regulated by zinc (Zhao et al. 2011; Smith and Loo 2012). In the view of the above results, zinc supplementation during the NSAID therapy seems fully reasoned.

The results of our study confirmed the advantage of the use of the zinc complexes and the combination of NSAIDs and ZHA over the parent drugs. The highest percentage of edema inhibition after single administration was achieved in rats receiving zinc–ibuprofen and zinc–naproxen, whereas the most meaningful results after triple administration were obtained for the physical mixtures of the parent NSAIDs and ZHA. The explanation for this is not simple. Certainly, zinc complexes and ZHA combinations with NSAIDs exert an uneven local effect on the gastric mucosa primarily because of the presence of a masked or free carboxyl group of the parent drug. Moreover, the differences in polarity, lipophilicity, and solubility presumably influence the absorption of the compounds from the gastrointestinal tract. Furthermore, the prolonged treatment may result in an adaptation of the gastric mucosa to specific drugs or combination of drugs, as was already suggested in the case of NSAIDs (Skeljo et al. 1992, 1996). Finally, the repeated ZHA administration may induce/enhance the synthesis/release/secretion of specific factors (proteins) involved in (anti)inflammatory processes (probably from the gastrointestinal tract). These issues should be examined in future studies.

Moreover, note the role of zinc deficiency in gastroduodenal ulcerogenesis during NSAID treatment. A zinc deficiency in the experimental animals amplified injuries and retarded the healing of ulcers (Watanabe et al. 1995; Lansdown et al. 2007). Studies revealed significantly increased oxidative damage and decreased Nrf2 expression in zinc-deficient mice (Zhao et al. 2011). Preparations containing zinc accelerated the healing of ulcers in the gastrointestinal tract, suppressed acid secretion, and improved the production of the tissue levels of metallothionein, mucus, and endogenous protective PGE_2_ (Bulbena et al. 1993; Tapiero and Tew 2003). According to the World Health Organization (WHO), zinc deficiency is one of the major life-threatening risk factors, particularly in developing countries and in the aging community in the industrial world (Eriksen et al. 2002). Moreover, impairment of zinc absorption is a documented concern of the long-term use of medications such as proton pump inhibitors, which are very often prescribed together with NSAIDs (Skrovanek 2014). Therefore, zinc supplementation may have an exceptionally beneficial effect on ulcer prevention and healing if there is an underlying zinc deficiency. The role of zinc supplementation during NSAID treatment to zinc-deficient patients should be examined in future studies.

## Conclusions

A lot of research is concentrated on the search for an ideal antiulcer drug, preferably of natural origin, which could be given prophylactically or therapeutically to patients during or after NSAID treatment. We chose the most frequently used NSAIDs, ibuprofen and naproxen, to synthesize their zinc complexes and determine their anti-inflammatory and ulcerogenic potential. The results of the hind paw edema test confirmed the validity of the combined use of zinc ions together with NSAIDs. In the case of a single administration, the highest percentage of edema inhibition was achieved in rats receiving zinc–ibuprofen and zinc–naproxen, whereas in the case of triple administration, the most meaningful results were obtained for the physical mixtures of parent NSAIDs and ZHA. Both NSAID complexes with zinc and their physical mixtures with ZHA reduced the severity of lesions as compared to the parent drugs. In the view of the above results, zinc supplementation during NSAID therapy may have a beneficial effect on ulcer prevention and healing by reducing the effective dose of the parent drug and increasing its potency. Nevertheless, defining the precise mechanism of the anti-inflammatory and gastroprotective effects induced by zinc ions in a free or complexed form during the NSAID treatment remains a topic for future research.
